# Oral Cancer Hazards Related to Tobacco Use and a Transtheoretical Model Assessment of Preparedness of Individuals With Oral Potentially Malignant Disorders to Quit Tobacco Use

**DOI:** 10.7759/cureus.48125

**Published:** 2023-11-01

**Authors:** Alpana Talukdar, Indrani Barman, Debojyoti Roy, Alaka Das, Putul Mahanta

**Affiliations:** 1 Dentistry, Nalbari Medical College and Hospital, Nalbari, IND; 2 Dentistry, Srimanta Sankaradeva University of Health Sciences, Guwahati, IND; 3 Dentistry, Fakhruddin Ali Ahmed Medical College and Hospital, Barpeta, IND; 4 Biochemistry, Assam Medical College, Dibrugarh, IND; 5 Biochemistry, Srimanta Sankaradeva University of Health Sciences, Guwahati, IND; 6 Forensic Medicine and Toxicology, Nalbari Medical College and Hospital, Nalbari, IND; 7 Forensic Medicine, Srimanta Sankaradeva University of Health Sciences, Guwahati, IND

**Keywords:** tobacco, transtheoretical model, risk factor approach, health risk, oral cancer

## Abstract

Objectives

There is a lack of knowledge on oral cancer and oral potentially malignant disorders (OPMDs) among the local communities of Northeast India. Since the habit of tobacco is linked to the culture and oral cancer is a widespread epidemic here, it is pertinent to assess the knowledge about risk factors and readiness to quit the habit among the study population.

Methods

The present research was done in the Tobacco Cessation Centre (TCC), Department of Public Health Dentistry (PHD) of Regional Dental College (RDC), Guwahati, Assam, from Jan 2023 to June 2023. This cross-sectional research was done among 200 patients aged 15-65 years. We have utilised the transtheoretical model (TTM) for assessing behaviour change. Oral inspection using a mouth mirror and explorer with adequate illumination was used to visualise the lesions. Data was collected through a self-administered questionnaire. Chi-square and odds ratio (OR) tests were used to see the association among the variables. The data was analysed with Microsoft Excel (Microsoft Corporation, Redmond, Washington) and IBM SPSS Statistics for Windows, Version 23 (Released 2015; IBM Corp., Armonk, New York, United States). A p-value less than 0.5 is considered to be statistically significant.

Results

Of the 200 participants, 64(32%) had some oral lesions linked to tobacco use. A significant correlation was observed between the oral lesions and TTM stages (OR=2.81, p<0.05). Among the participants, most were aware only of tobacco (68%) and quid chewing (58%) practices as risk reasons for developing oral cancer.

Conclusion

The study participants' awareness of OPMDs, their health risks, and clinical signs and symptoms could be higher. A significant relationship was seen between OPMDs and TTM behavioural stages.

## Introduction

Oral and pharyngeal cancers are a dominant source of illness and death, with an annual global estimated incidence of about 3,77,713 for oral and 98,412 for oropharyngeal cancers, causing 1,77,757 deaths in 2020, excluding salivary or malignant tumours of the nasopharynx and the pyriform fossa. Developing nations account for two-thirds of these cancer cases [1].

The incidence of mouth and oropharynx malignancies in India standardised to the world standard population in 2003 was 12.6 per 100,000 [2]. The oral malignancy ranked third in India and sixth among all malignancies worldwide. Yet, there is still a lack of public knowledge of this severe disease and the variety of oral potentially malignant disorders (OPMDs) that frequently precede it, particularly on the Indian subcontinent and among immigrants from the region. This also applies to developed countries in comprehending the widely recognised risk factors [3]. The delay in diagnosing OPMDs is likely because of no general insight into the indicators, symptoms, and causal factors. There needs to be expertise for early identification by healthcare providers. Because premalignant lesions trigger most oral cancer and are frequently asymptomatic, routine dental screening is critical for early detection.

The transtheoretical model (TTM) is an often-used methodology in behavioural change modelling, which states that changing a habit is not coincidental but rather a process in which different people are at various phases in modification and preparation [4]. According to the TTM, when altering a behaviour consciously, individuals go through a series of stages and progress through stage-specific modification preparations, which are changed by levels of decisional balance and self-efficacy [5]. Various studies have documented the effectiveness of the TTM in preventing interferences for chronic illnesses, such as diabetes and cancer [6]. The evidence for the TTM's applicability in tobacco cessation and prevention is strong and expanding [7]. Tobacco smoking is ingrained in Indian culture and widespread among the northeast Indian population. As oral cancer is a widespread epidemic in this region, it is essential to assess the knowledge regarding the risk factors and readiness to quit the habit among individuals.

Also, ordinary people must be conversant with its early signs and risk factors to lower the suffering and death due to oral cancer. Nonetheless, there is very little data on the point of awareness in Northeast India. Additionally, not many studies investigate the relationship between behavioural stages and preparedness to give up tobacco use.

Hence, the present research planned to assess the cognisance of oral malignancy and OPMDs, their initial symptoms and risk determinants of cases attending RDC in northeast India. Additionally, the study sought to evaluate the preparedness to give up the substance tobacco use amongst cases with OPMDs visiting the study centre using the TTM. We postulated that the existence of OPMDs would affect patients' willingness to give up tobacco products.

## Materials and methods

This cross-sectional investigation was conducted in the Tobacco Cessation Centre (TCC), Department of Public Health Dentistry (PHD), Regional Dental College (RDC), Guwahati, Assam. The study has included patients referred from the Outpatient Department and patients referred from camps conducted in rural parts of Assam and district hospitals. Patients aged 15-65 years were included in the current study.

Because of scanty information on oral cancer and OPMDs among the local communities, a brief investigation in regions distinct from the main study but related to it was carried out to evaluate the prevalence of disease, risk factors, and non-response rate so that the sample size might be estimated with adequate power. Thus, the sample size required for the main study was fixed at 200 participants, considering the prevalence of oral cancer as 12.6% and a non-response rate of 10% with a 95% confidence interval.

Data were collected using a self-administered questionnaire consisting of three parts: sociodemographic details, questions on risk factors and behaviour change. The behavioural change was assessed using the TTM consisting of four different stages. Individuals in the pre-contemplation stage have no plans to give up the habit within the next six months. Individuals in the contemplation stage intend to give up tobacco within the next six months. Similarly, those intending to give up the habit within the next thirty days are at the preparation stage. Individuals who have quit tobacco for the last six months were at the action stage. Oral inspection using a mouth mirror and explorer with adequate illumination was conducted to detect the presence or absence of a lesion or OPMD.

Statistical assessment

After data collection, proforma were checked for incompleteness, faults or trivialities. The data was analysed with the help of Microsoft Excel (Microsoft Corporation, Redmond, Washington) and IBM SPSS Statistics for Windows, Version 23 (Released 2015; IBM Corp., Armonk, New York, United States). Descriptive statistics were used to summarise both quantitative and qualitative data. The Chi‑square test was done to examine the relationships between two categorical variables, and the odds ratio (OR) was computed. To check the connection between behaviour change and OPMD, data on OPMD were dichotomised based on the existence or non-existence of oral lesions. Similarly, TTM stages were dichotomised by combining pre-contemplation and contemplation into one group and preparation and action into another. A p-value less than 0.5 is considered to be statistically significant.

The institutional review board of RDC, Guwahati, has given the ethical approval to conduct this research vide ref: RDC/29/2011/1556 and done as per the guidelines of the Helsinki Declaration, as revised in 2013.

## Results

The study comprised 200 participants, 118 (59%) males and 82(41%) females. Most participants were aged 21-50 years. Of the 200 participants, 32 reported no formal education, while most male participants were in primary school (44.1%). The education status was found to be notably varied between genders. The onset age and tobacco exposure ranged from 10-52 years (mean age 25.4 ± 6.3 years) and 1- 45 years (mean age 12.2 ± 10.7 years). The duration of tobacco habit was above 15 years among most participants (Table 1).

**Table 1:** Characteristics of the participants n: number of the cases

Variables	Categories	Male (n=118)	Female (n=82)	p-value for the Chi-Square test
Age	10-20	8 (6.8%)	6 (7.3%)	0.89
	21-30	22 (18.6%)	10 (12.2%)	
	31-40	29 (24.6%)	23 (28.0%)	
	41-50	31(26.3%)	24 (29.3%)	
	51-60	18 (15.2%)	12 (14.6%)	
	61-70	10 (8.5%)	7 (8.5%)	
Level of education	Illiterate	20 (16.9%)	12 (14.6%)	0.009
	Primary school	52 (44.1%)	24 (29.3%)	
	Middle School	12 (10.2%)	20 (24.4%)	
	High School	6 (5.1%)	12 (14.6%)	
	Diploma	18 (15.2%)	8 (9.8%)	
	Graduate	10 (8.5%)	6 (7.3%)	
Duration of tobacco use	6 months-5 years	8 (6.8%)	3 (3.6%)	0.74
	6-10 years	22 (18.6%)	15 (18.3%)	
	11-15 years	36 (30.4%)	29 (35.4%)	
	>15 years	52 (44.1%)	35 (42.7%)	
Oral lesions	Absent	69 (58.5%)	67 (81.7%)	<0.001
	Present	49 (41.5%)	15 (18.3%)	

**Figure 1:**
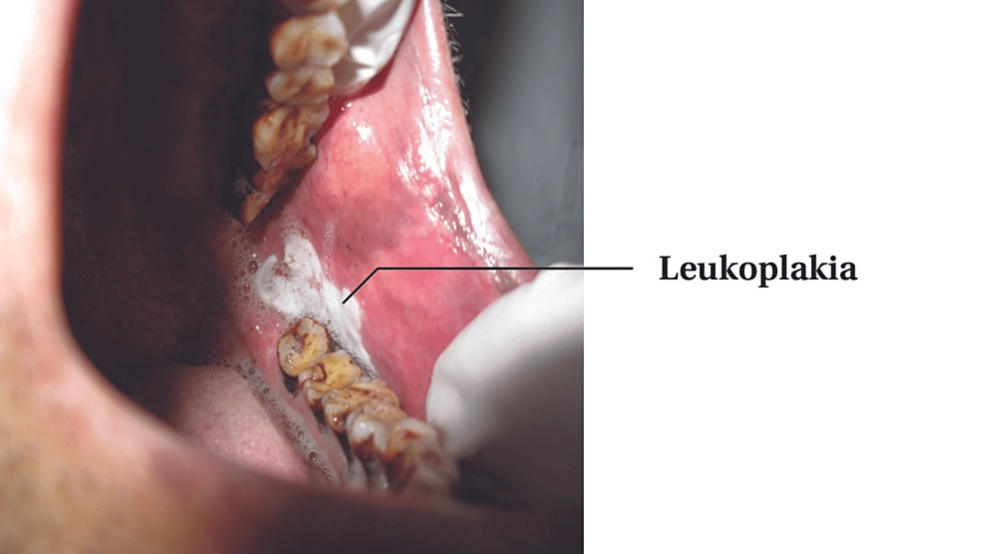
Leukoplakia

**Figure 2:**
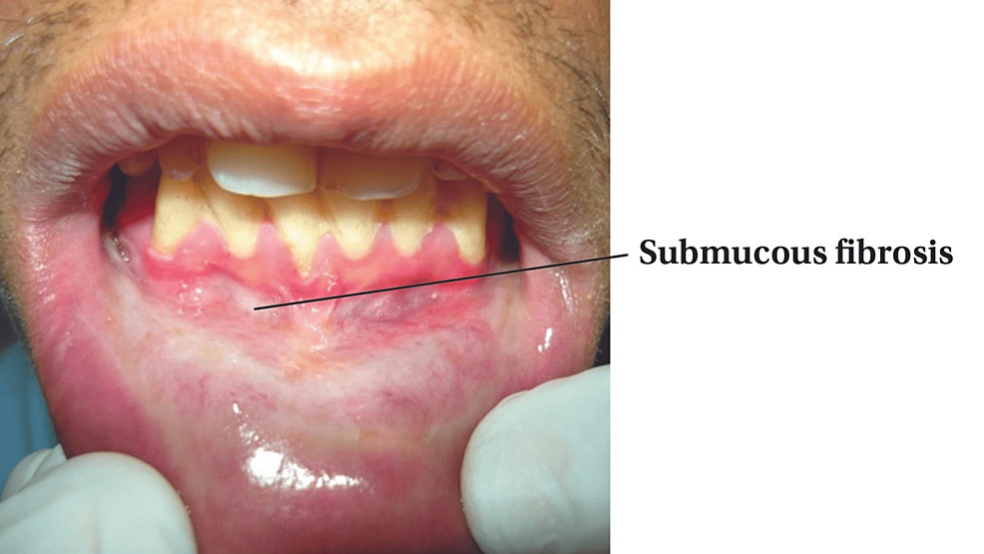
Oral submucous fibrosis

Out of the 200 participants, 64(32%) had some oral lesions associated with tobacco use, among whom 49 were male and 15 were female (p-value<0.001). The lesions found included smoker's palate, leukoplakia (Figure 1), oral submucous fibrosis (Figure 2) and erythroplakia.

The TTM shows that most participants (41%) were in the contemplation stage, among whom 70 (35%) were aware of oral cancer and OPMDs as the worst outcome. Only 11 (5.5%) participants were in the action stage, and all were aware of the potential risk factors of the diseases under study (Table 2).

**Table 2:** Distribution of participants based on awareness of tobacco-related risk of oral cancer and OPMDs and transtheoretical model behavioural stages OPMDs: Oral potentially malignant disorders

Behavioural stage	Total	Aware	Unaware	p-value for the Chi-Square test
Pre-contemplation	41(20.5%)	28(14%)	13(6.5%)	0.03
Contemplation	82(41%)	70(35%)	12(6%)	
Preparation	66(33%)	56(28%)	10(5%)	
Action	11(5.5%)	11(5.5%)	0	

Phases of the TTM and oral illnesses were shown to be strongly related to OR = 2.45 (p-value<0.01, CI: 1.33-4.51). Those with tobacco-related oral illnesses were more prevalent in the preparation and action stage (Table 3).

**Table 3:** Relationship among OPMDs and TTM behavioural stages OPMDs: Oral potentially malignant disorders; TTM: transtheoretical model; *: p<0.05 (statistically significant); OR: unadjusted odds ratio CI: confidence interval

OPMD	Preparation + action	Precontemplation + contemplation	p	Chi-Square	OR (95% CI)
Present	34	30	0.003	8.52	2.45 (1.33-4.51)
Absent	43	93			

Among the participants, most were only aware of tobacco (68%) and quid chewing habit (58%) as a risk factor for developing oral cancer. Over 80% of the participants were negligent of alcohol, nutritional deficiency and inadequate oral cleanliness as potential factors for developing OPMDs/oral cancer (Table 4).

**Table 4:** Distribution of knowledge on the risk association of tobacco use with oral malignancy and OPMDs OPMDs: Oral potentially malignant disorders

Awareness of risk factors	Not aware	Aware of only oral cancer	Aware of oral malignancy and OPMDs
Betel quid chewing	32%	58%	10%
Smoking	64%	29%	7%
Alcohol	82%	14%	4%
Nutritional deficiency	84%	12%	4%
Poor oral hygiene	80%	16%	4%
Tobacco	23%	68%	9%
Awareness of symptoms	84%	13%	3%

## Discussion

The absence of common knowledge of the symptoms, indications, and risk factors is thought to cause a delay in the diagnosis of OPMDs [8]. The best strategy for enhancing survival to minimise the risk of complications, deformity, length of medical care, and treatment expenditure is identifying oral cancer early. The time between the patient first discovering a sign or symptom and their initial consultation with a healthcare practitioner leads to patient delay. Professional delay is the interval between the patient's initial visit with the healthcare provider and the definite diagnosis. The diagnostic delay, comprising either patient or professional delay or both, has recently been identified as an essential factor that reduces survival and degrades treatment outcomes [8].

Although most participants were aware of oropharyngeal cancer, an alarming lack of awareness of OPMDs was observed in the current study. The findings agree with a similar study [9]. Numerous research studies revealed a lack of participant knowledge about oral malignancy [10,11]. Most oral malignancy cases are still only investigated in their advanced stages. Often, asymptomatic premalignant lesions typically cause oral cancer. As these lesions in the initial stage do not affect regular masticatory functions, patients frequently ignore them because of insufficient awareness about the illnesses. The delay in identifying OPMDs also results from an insufficient understanding of early detection by healthcare providers [9,12].

Young people and adolescents are most likely to use tobacco products [13-17]. The typical age of tobacco initiation in the current research was 25.4 ± 6.3 years. As per the global adult tobacco survey (2010), the overall mean age for starting a tobacco habit in India was 17.8 years among all ages, which is lower than the current findings [18]. About 4/5th of the participants started the habit of tobacco early in their 30s. Another recent study in northeast India reported that over 90% of alcohol and tobacco users between 10-30 years agree with our results [19]. As per the GATS 2 survey, most participants used tobacco aged between 25 and 44 years [20].

Most of the respondents had a low level of education, with around sixteen percent of the overall participants required to be literate in the current study. Education, regardless of the form of usage, is a significant essential influence on tobacco use [21-23]. The lack of education, peer pressure and the need for more awareness lead to early initiation of tobacco use.

According to the TTM, approximately one-half of all participants were in the contemplation stage, followed by preparation, pre-contemplation, and action stages. Ambivalence traverses into the development of pre-contemplation and contemplation stages. Individuals in these stages are either oblivious to harmful behaviour or hesitant to change it [4]. They need more confidence in changing their behaviour [24]. At the same time, individuals in the preparation stage are motivated to quit [25]. People in the preparatory and action stages of cessation require various approaches than those in pre-contemplation or contemplation stages [26]. There was a substantial relationship between OPMDs and TTM behavioural stages. Participants with OPMDs were inclined three times more to advance from pre-contemplation and contemplation to preparation and action. Thus, those who know the existence and implications of potentially malignant illnesses are more motivated to quit tobacco.

The present research analysis reveals that knowledge about oral malignancy and OPMDs is low in this part of India. Over 90% of those who participated in the present study needed to be made aware of the risk factors and clinical manifestations of OPMDs. Oral cancer survival rates are pretty low, at around 50% overall, and have not improved significantly in recent decades despite breakthroughs in therapeutic measures. Quickly revealing oral malignancy is the most effective way of lowering the disease's fatality, morbidity, and deformity rates [27]. A lack of understanding and knowledge regarding the indications, manifestations, and associated risks frequently results in a diagnostic delay in detecting PMDs. Preventing and detecting OPMDs early can lower the incidence and improve the survival of those who acquire oral malignancy.

The present study delivers an essential intuition into the strong relationship between TTM behavioural stages and OPMD risk variables. Betel nut and tobacco chewing are integral to Assamese culture [28]. Being the only dental college in the state from 1982 until 2018, patients visited the study hospital from all parts of Assam. Thus, the study population gives a fair representation of the current pattern of tobacco use in Assam.

Although tobacco consumption substantially correlates with socioeconomic status, the research on how cessation varies by socioeconomic characteristics still needs to be more conclusive, particularly in developing nations [29]. For tobacco control, policymakers should consider the socioeconomic patterning of tobacco consumption. Adolescents must be targeted to help them overcome peer impact and avoid practising hazardous habits. Since no single healthcare provider has access to all tobacco users and prospective future users, it is vital that medical authorities, like dental professionals, actively participate in the fight against tobacco.

Limitations

As the current research is an observational study conducted on patients visiting the dental college for treatment, it has a limitation in its generalizability. The used proforma may suffer from self-reporting biases. Future research is necessary to address these limitations.

## Conclusions

Research has found that knowledge about oral malignancy and OPMDs is low in India's northeastern areas. This finding has been replicated in numerous global studies, notably on initial signs and indicators, risk factors, and prevention methods. The presence of OPMDs and TTM behavioural stages were found to be strongly associated. Patients with OPMDs showed a higher level of readiness to give up tobacco. Dental professionals could progress towards reducing this hazardous practice by better understanding the behavioural stages of TTM and factors associated with tobacco use.

Raising awareness is essential among the exposed people. In India, there is an urgent need for ongoing national education and health promotion initiatives emphasising oral cancer while integrating these messages with broader health messaging and employing a common risk factor approach. These must consider the gaps in knowledge and the preferred or available communication routes for the diverse communities.
